# Clinically Significant Infections in Surgical Mortality

**DOI:** 10.1111/ans.70780

**Published:** 2026-06-04

**Authors:** Emily‐Rose Lodge, Helena S. Kopunic, Nathan E. K. Procter, Sivakumar Gananadha

**Affiliations:** ^1^ Australian Capital Territory Audit of Surgical Mortality (ACTASM) Royal Australasian College of Surgeons Australia; ^2^ School of Medicine and Psychology Australian National University Canberra Australia

**Keywords:** clinically significant infections, hospital‐acquired infections, infectious organisms, quality of care, surgical mortality, surgical site infections, surgical specialty

## Abstract

**Introduction:**

Clinically significant infections (CSIs) are a common complication identified in cases of surgical mortality, associated with prolonged hospital stays, unplanned escalation of care and increased risk of death. This study aimed to examine the burden of CSIs in surgical deaths in the Australian Capital Territory and identify contributing factors and potential opportunities to improve surgical outcomes.

**Methods:**

The Australian Capital Territory Audit of Surgical Mortality (ACTASM) collects data on in‐hospital surgical mortality. This report compared cases with and without CSIs from 1 January 2015 to 31 December 2024.

**Results:**

Of 1089 cases, 363 had CSIs. Cases with CSIs had a higher proportion of comorbidities and longer lengths of stay but underwent fewer procedures. More patients acquired infections during than before admission. Sepsis was the most common infection acquired before admission, whereas pneumonia was the most common infection acquired during admission. General surgery had the highest number of CSIs, while urology had the highest proportion of CSIs relative to case volume. Rates of unplanned readmissions to hospital, unplanned admissions to critical care unit, unplanned returns to theatre and postoperative complications were higher in cases with CSIs.

**Conclusion:**

Surgical patients with CSIs who died had more comorbidities and longer lengths of stay but were less likely to undergo an operation than those without CSIs. Unplanned events were higher in patients with CSIs. These findings underscore the significant impact of CSIs on patient outcomes, highlighting the need for effective preventive strategies and timely management to optimise patient outcomes.

## 1 Introduction

Clinically significant infections (CSIs) are a major contributor to morbidity and mortality among surgical patients. They complicate the perioperative course, increase physiological stress in already vulnerable patients, and frequently precipitate a cascade of adverse events including sepsis, organ failure and death. Data from the Australia and New Zealand Audit of Surgical Mortality (ANZASM) indicate that nearly one‐third of patients who die in surgical care have a CSI [1]. These infections may be present prior to admission and related to the underlying disease, or acquired during hospitalisation as a consequence of surgery, critical illness, or prolonged exposure to healthcare environments.

Hospital acquired infections (HAI) are among the most common complications of in‐patient care. HAI are associated with prolonged hospital stays, increased healthcare costs, higher rates of unplanned critical care unit admissions, and higher rates of morbidity and mortality [2, 3] Surgical patients are particularly susceptible to HAIs due to the traumatic nature of surgery which results in tissue injury, foreign material implantation, exposure to invasive devices and immunosuppression [4, 5, 6, 7, 8, 9]. Considerable attention has been directed toward preventing surgical‐site infections, while the broader spectrum of CSIs—including pneumonia, sepsis and bloodstream infections—remains an ongoing challenge in surgical populations.

ANZASM plays a critical role in improving patient outcomes by systematically reviewing all in‐hospital deaths that occur under surgical care and analysing for clinical and system‐level issues. ANZASM provides targeted feedback to surgeons, hospitals and healthcare networks to improve surgical care and patient safety across Australia.

The Australian Capital Territory Audit of Surgical Mortality (ACTASM), a participating partner in ANZASM, collects data on surgical mortalities including the presence and characteristics of CSIs. We undertook a review of all surgical deaths in the Australian Capital Territory (ACT) over a 10‐year period to examine the nature and burden of CSIs. This study aims to identify contributory factors and highlight lessons that may inform strategies to reduce infection‐related harm and improve surgical outcomes by comparing cases with and without CSIs, and exploring infection type, timing, specialty distribution and associated unplanned events.

## 2 Methods

ACTASM is an initiative of the Royal Australasian College of Surgeons that is supported by the ACT Department of Health. We reviewed surgical deaths from public and private hospitals across the ACT that were reported to ACTASM. There is full participation of all surgeons and health facilities in the ACT. The ACTASM evaluation process includes all incidents of in‐hospital mortality where the patient was admitted by a surgeon, regardless of whether a surgical procedure was performed, or a surgical procedure was performed by a surgeon during a non‐surgical admission. Cases were excluded if the patient was admitted for terminal care, incomplete data, or if the cases were still undergoing evaluation as of census date (10 February 2025). ACTASM receives notification of patient deaths from all hospitals in the ACT. Following notification to ACTASM of a death, ACTASM notifies the treating surgeon that a death has occurred while the patient was under their care. The responsible surgeon provides details of the patient's hospital admission in a surgical case form that is then deidentified and sent for first‐line assessment by an independent surgeon in the appropriate specialty. This assessment either reaches a conclusion regarding the quality of care resulting in closure of the case or refers the case for a second‐line assessment (which includes medical note review) if further information or clarification is required. Assessors are asked to identify any clinical management issues and aspects of management that may have contributed to death and are separate to the underlying disease process. Each of the clinical incidents identified is classified as either: [1] *Area for consideration*: An aspect of care *could* have been better; [2] *Area of concern*: An aspect of care *should* have been better; or [3] *Adverse event*: An aspect of care contributed to or caused death. In addition, assessors are asked to indicate the perceived level of impact and preventability of the incident. A case is closed when feedback has been returned to the surgeon.

Unless otherwise specified, analyses in this study were carried out by comparing data between two cohorts, patients with a clinically significant infection and patients without a clinically significant infection. Data analysed for this study included patient demographics, length of stay, patient comorbidities (including advanced malignancy, age, alcohol use, cardiovascular, diabetes, hepatic, neurological, obesity, renal, respiratory, tobacco use and ‘other’), American Society of Anesthesiologists (ASA) physical status [4] classification score, details of the infections (including timing of infection acquisition, the type of infection, microbiological data and the appropriateness and timing of infection treatment), surgical specialty of the treating surgeon, operative details (including the number of operations performed, the timing of operations and consultant presence in theatre), unplanned events (including postoperative complications, unplanned returns to theatre, unplanned readmissions to a critical care unit and unplanned readmissions to hospital), appropriateness of the treatment, speciality details and any clinical management issues. Data were extracted on 10 February 2025 and included closed cases in which patient deaths that occurred from 1 January 2015 to 31 December 2024. This study was approved by the ANZASM Committee and was undertaken as part of its quality assurance activities.

Data analyses were carried out in Microsoft Access, Excel, RStudio and GraphPad Prism 10.5.0 (Massachusetts, USA). Differences between categorical variables were analysed via the *χ* ^2^ test, and differences in continuous variables were analysed via the independent *t*‐test or Mann–Whitney U test, as appropriate.

A multivariable logistic regression model was constructed with the presence of clinically significant infection as the dependent variable. Variables with clinical relevance and/or univariate association were included in an initial full model. Backward stepwise selection based on Akaike Information Criterion was performed to identify independent predictors of CSI. Adjusted odds ratios with 95% confidence intervals are reported for values with *p* < 0.05.

## 3 Results

Between 1 January 2015 and 31 December 2024, 1369 deaths underwent the ACTASM evaluation process. A total of 88 cases were excluded from analysis because they were still undergoing evaluation as of the census date or involved patients admitted for terminal care (*n* = 188). Four cases with unknown infection status were also excluded from analysis. The remaining 1089 cases were included in the study and were stratified into two cohorts: those with a CSI (*n* = 363) and those without a CSI (*n* = 726). Patient characteristics for both groups are summarised in Table 1. The length of stay for CSI cases was almost double that of non‐CSI cases (9 days versus 5 days, *p* < 0.001) (Table 1).

**TABLE 1:** Patient demographics.

Characteristic	Cases with a CSI (*n* = 363)	Cases without a CSI (*n* = 730)	*p*
Age, median years (IQR)^a^	77 (68–84)	75 (63–83)	< 0.001
Sex, male: Female, %	58.1: 41.9	57.7: 42.3	0.8
Admission type, elective: Emergency, %	17.9: 82.1	13.4: 86.5	0.048
Length of stay, median days (IQR)^a^	9 (3–21)	5 (2–11)	< 0.001
Operation performed, %	77.4	81.1	0.15
Perceived risk of death, % (surgeon)^b^			
Minimal	3.6	3.9	0.80
Small	13.5	13.1	0.85
Moderate	29.9	24.6	0.10
Considerable	44.8	47.0	0.54
Expected	7.8	10.2	0.27
Not recorded	0.4	1.2	0.23
Comorbidity present, %^c^	93.7	77.1	< 0.001
Type of comorbidity, %*			
Advanced malignancy	21.8	16.9	0.054
Age	63.9	48.5	< 0.001
Alcohol use	0.8	0.8	1.00
Cardiovascular	59.0	51.9	0.028
Diabetes	24.0	16.5	0.0032
Hepatic	7.7	4.5	0.032
Neurological	20.1	21.8	0.53
Obesity	12.1	9.2	0.14
Other	23.1	19.0	0.11
Renal	28.1	18.7	< 0.001
Respiratory	38.0	24.8	< 0.001
Tobacco use	1.1	0.6	0.32

Abbreviations: CSI, clinically significant infection; IQR, interquartile range.

^a^ 25th and 75th percentiles of interquartile range.

^b^ Overall mortality risk as determined by treating surgeon if an operation was performed.

^c^ Proportion of cases with at least one comorbidity.

* *p* value < 0.05, indicating a statistically significant difference between the two cohorts.

An operation was performed in 77.4% of CSI cases, compared with 81.1% of non‐CSI cases (*p* = 0.15). Patients with a CSI had a significantly higher burden of comorbidities than those without a CSI (93.7% versus 77.1%; *p* < 0.001). Cardiovascular disease and advanced age were the most common comorbidities in both cohorts. Patients with a CSI had a higher proportion of every type of comorbidity except for neurological disease, with the most notable differences observed in age‐related comorbidity (15.4% higher in the CSI group, *p* < 0.001) and respiratory disease (13.2% higher in the CSI group, *p* < 0.001).

A difference in the distribution of the American Society of Anesthesiologists (ASA) physical status classification scores was observed, driven mainly by a higher proportion of patients without a CSI being classified as ASA 5 compared with patients with a CSI (Table 2). Even though there was a trend toward a lower ASA grade in CSI cases, most patients in both cohorts were classified as ASA 4, indicating severe systemic disease.

**TABLE 2:** ASA grade.

ASA grade *n* (%)	Cases with a CSI *n* = 363	Cases without a CSI *n* = 730	*p‐value*
1	1 (0.3)	11 (1.5)	0.056
2	21 (5.8)	40 (5.5)	0.70
3	106 (29.2)	188 (25.8)	0.95
4	167 (46.0)	290 (39.7)	0.90
5	38 (10.5)	141 (19.3)	0.0062
6	4 (1.1)	18 (2.5)	0.93
Unknown	26 (7.2)	42 (5.8)	0.077

Abbreviations: ASA grade, American society of anesthesiologists physical status classification system; ASA I, a normal healthy patient; ASA II, a patient with mild systemic disease; ASA III, a patient with severe systemic disease; ASA IV, a patient with severe systemic disease that is a constant threat to life; ASA V, a moribund patient who is not expected to survive without the operation; ASA VI, a declared brain‐dead patient whose organs are being removed for donor purposes.

### 3.1 Infection Details

**FIGURE 1:**
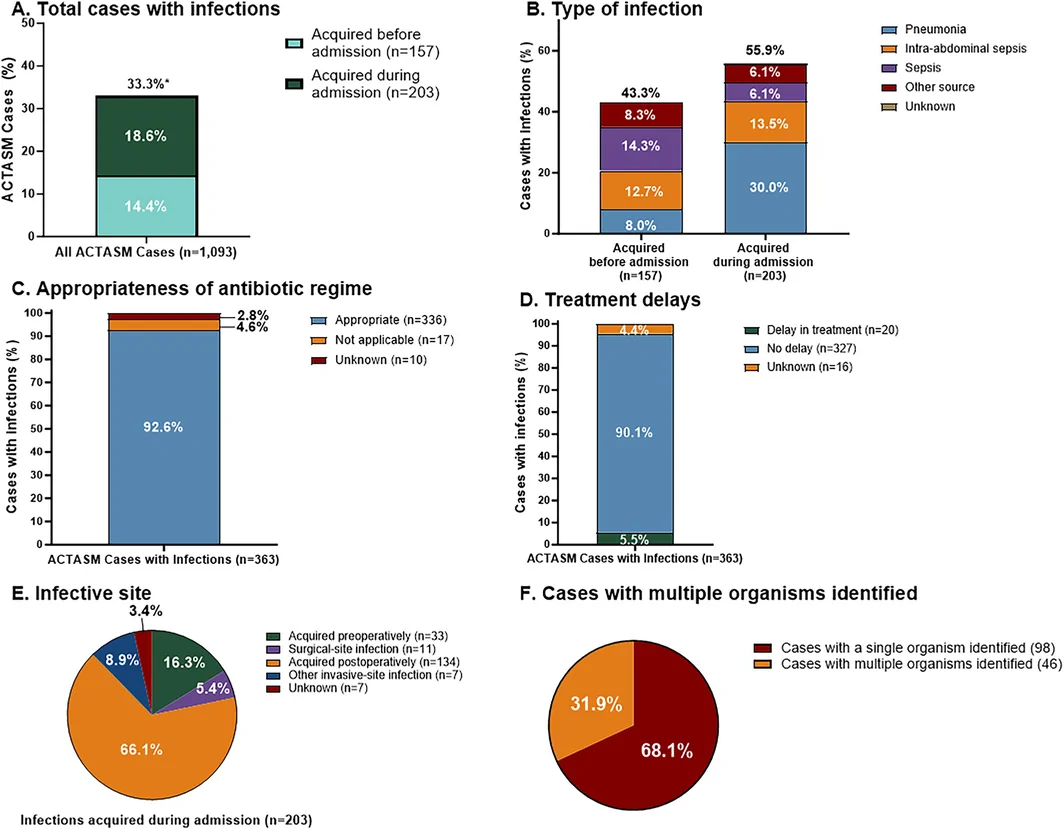
Clinical characteristics of CSIs in ACTASM cases. Abbreviations: ACTASM, Australian capital territory audit of surgical mortality; CSI, clinically significant infection.

**FIGURE 2:**
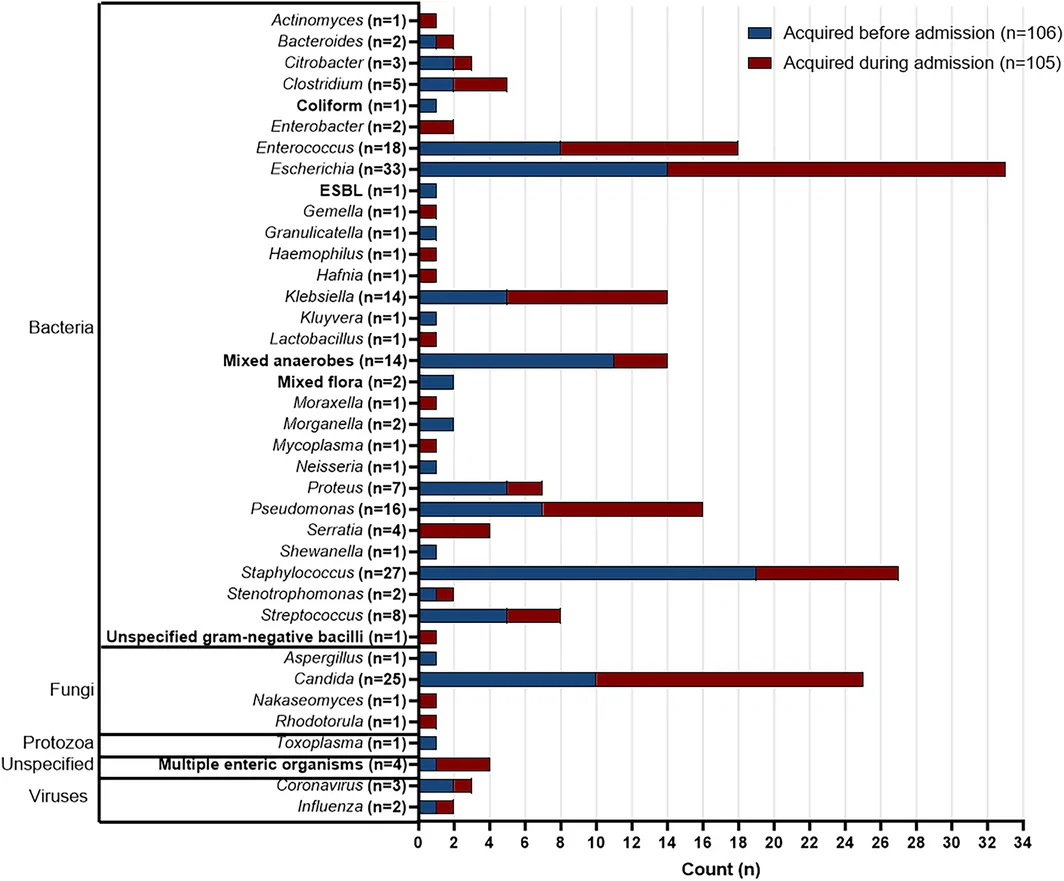
Infectious organisms identified in cases with CSIs by time of infection. Acronym: ESBL, extended‐spectrum beta‐lactamase.

During the study period, 33.3% of cases (363 out of 1089) had a CSI. A higher proportion of these infections were acquired during admission rather than being present on admission (55.6% versus 43.3%, *p* = 0.02, Figure 1A). The most common type of CSI acquired before admission was sepsis, while the most common type acquired during admission was pneumonia (Figure 1B). The antibiotic regimen was deemed appropriate in most cases (97.2%) (Figure 1C). In addition, treatment was initiated without delay in 90.1% of CSI cases (Figure 1D). A substantial proportion (66.0%) of infections acquired during admission were postoperative in origin (Figure 1E). The infective organism was identified in 39.6% (*n* = 144) of cases where a CSI was reported. Microbiological data revealed that 68.1% (*n* = 98) of these cases involved a single infective organism (Figure 1F). Figure 2 shows the different infectious organisms identified in cases with CSIs by time of infection.

### 3.2 Surgical Specialty

**FIGURE 3:**
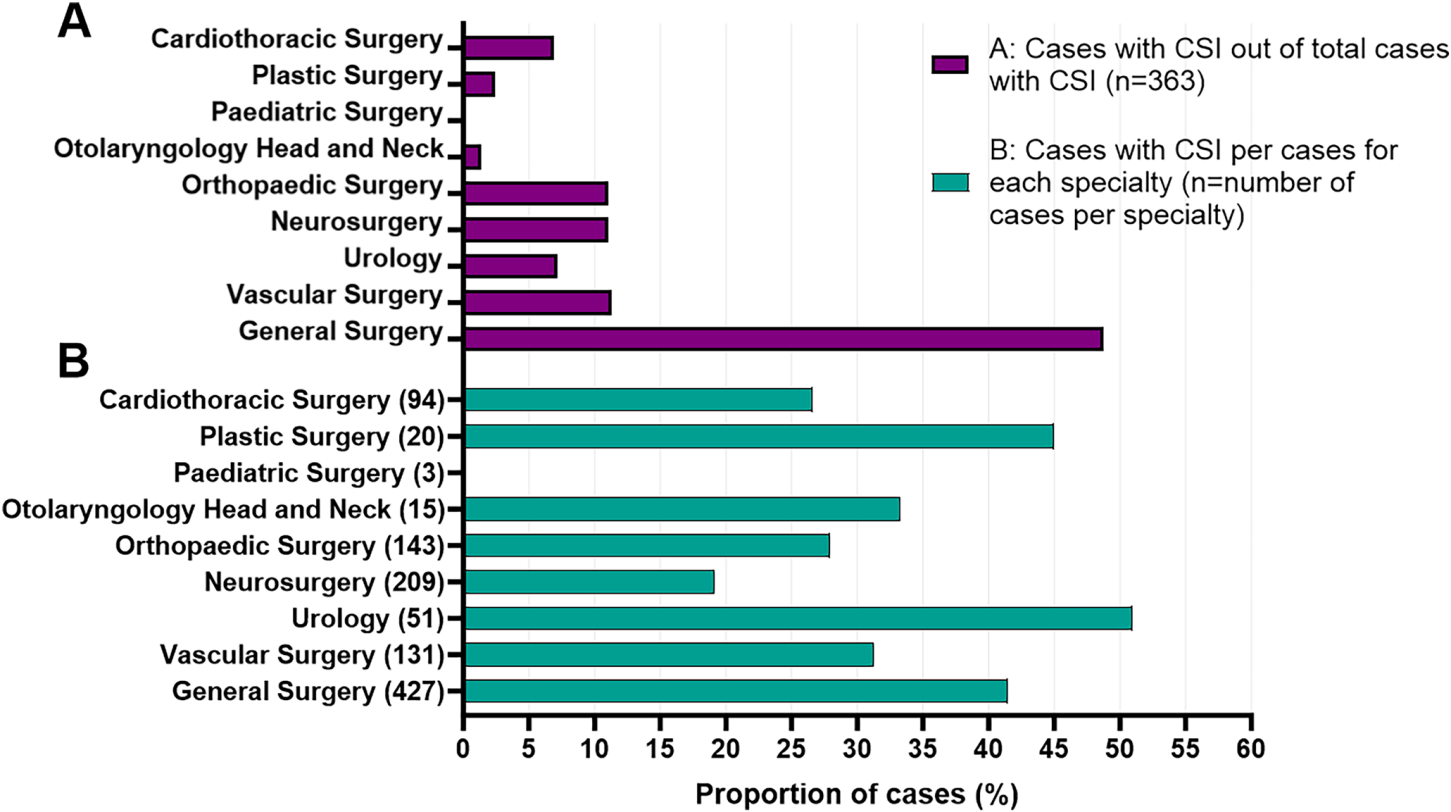
Surgical speciality of cases in which a CSI was identified. Abbreviation: CSI, Clinically significant infection.

General surgery patients accounted for the highest number of CSI‐related deaths, representing 48.8% (177 out of 363) of all CSI mortalities; more than three times higher than what was observed for any of the other surgical specialties (Figure 3A). CSIs were identified in over half of all urology cases (51.0%; 26 out of 51) and in 45.0% (9 out of 20) of plastic surgery cases (Figure 3B), indicating that these specialties carry a high infection burden relative to their case volume.

### 3.3 Operations

**FIGURE 4:**
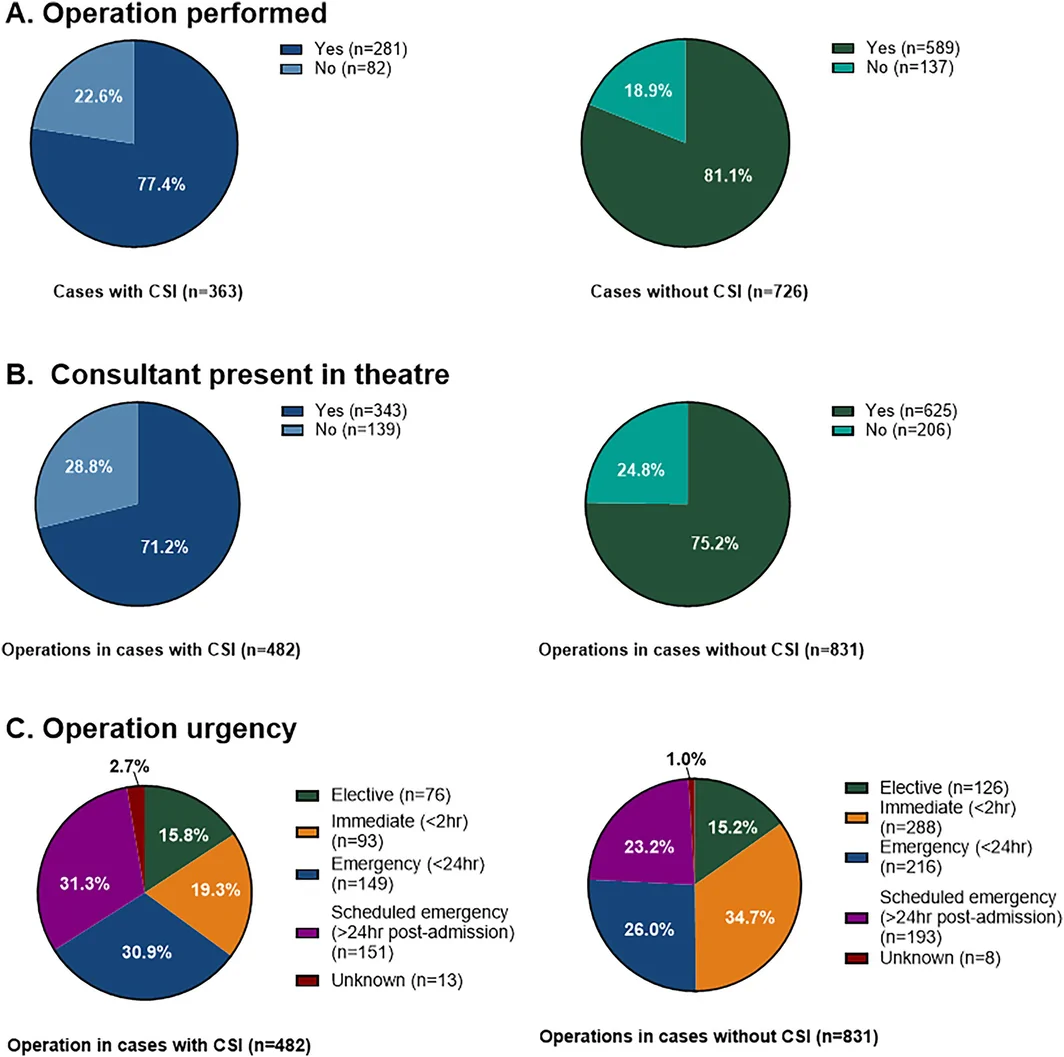
Operation characteristics of cases by CSI. Abbreviations: CSI, clinically significant infection; hr., hours.

A slightly higher proportion of patients without a CSI underwent surgery and had a consultant surgeon present in theatre—whether operating, assisting or present—compared with patients with a CSI (81.1% versus 77.4%, *p* = 0.15 and 75.2% versus 71.2%, *p* = 0.62) (Figure 4A and Figure 4B). The timing and urgency of surgery differed significantly between the two cohorts, with patients with a CSI being significantly less likely to have an immediate operation and significantly more likely to have a scheduled emergency operation than the cohort without a CSI (*p* < 0.001 Figure 4C). Over a third of the operations in cases without a CSI were performed immediately (within 2 h of admission), compared with less than a fifth in cases with a CSI (34.7% versus 19.3% *p* < 0.001). Most operations in patients with a CSI occurred more than 24 h after admission (31.3%).

### 3.4 Unplanned Events

**FIGURE 5:**
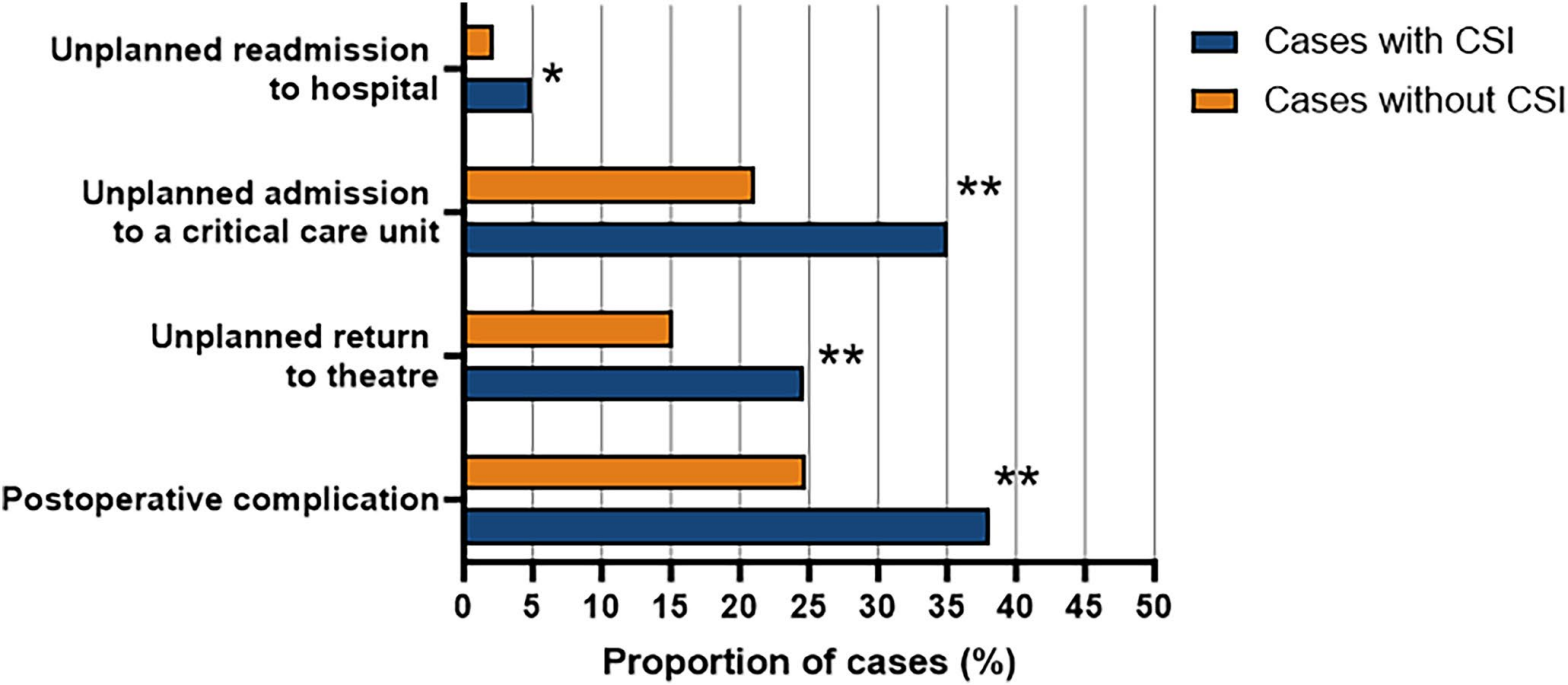
Unplanned events according to presence of CSI. Abbreviation: CSI, clinically significant infection. *p < 0.05; **p < 0.01.

Unplanned events include postoperative complications, unplanned returns to theatre, unplanned readmissions to a critical care unit and unplanned readmissions to hospital. Cases with a CSI experienced a higher proportion of all four unplanned events than did cases without a CSI (Figure 5). Statistically significant differences were observed between the CSI and non‐CSI groups across all categories of unplanned events, indicating a consistent pattern of increased adverse outcomes among patients with an infection (Figure 5).

**FIGURE 6:**
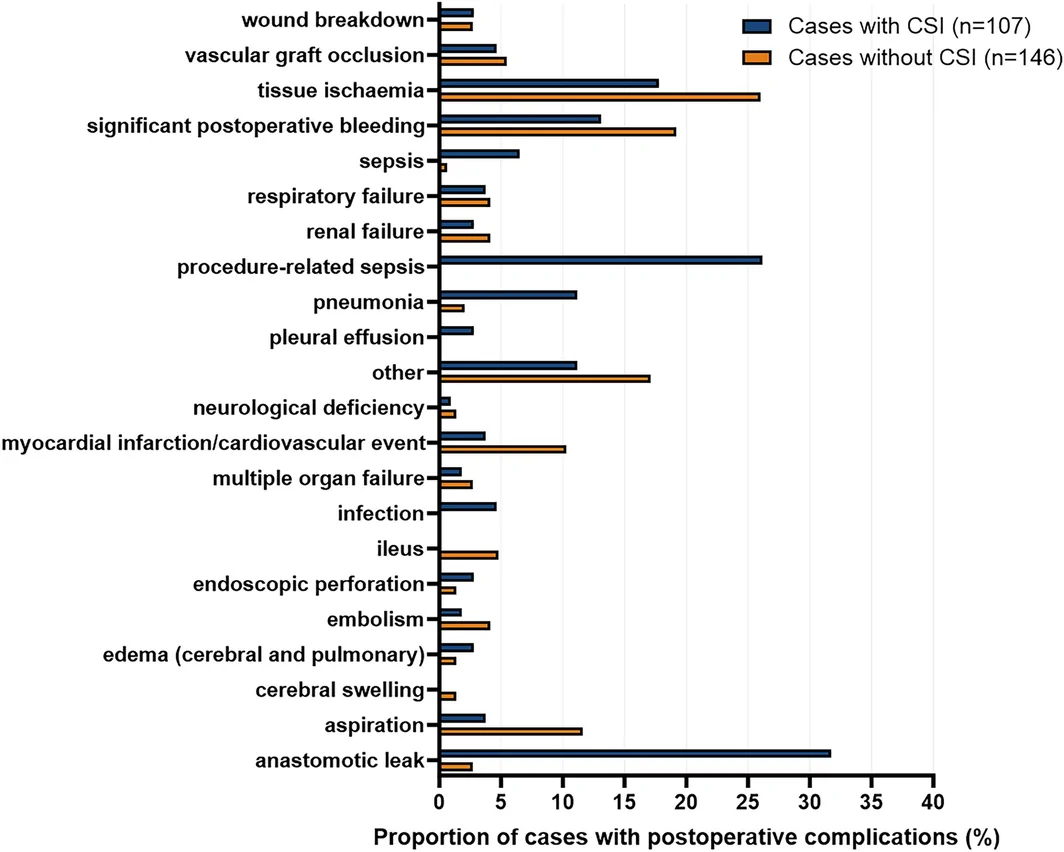
Postoperative complications according to presence of CSI. Abbreviation: CSI, clinically significant infection.

Postoperative complications are detailed further in Figure 6.

### 3.5 Multivariate Analysis

Those factors that were significantly different between CSI and non‐CSI groups on univariate analysis were subject to multivariate regression (ASA grade, presence of comorbidities, admission type, surgical specialty, occurrence of postoperative complications, unplanned returns to theatre, unplanned admissions to ICU and unplanned readmissions to hospital). Surgical specialty, comorbidity burden, unplanned returns to theatre and unplanned admission to ICU survived the analysis, admission type (emergency vs. elective) trending towards significance (Table 3). Follow up *χ* ^2^ analysis of surgical specialties confirmed a significant difference in distribution between CSI and non‐CSI cohorts (*p* < 0.001) with the difference being driven largely by General Surgery and Neurosurgery. Urology did not achieve statistical significance (*p* = 0.10). Comorbidity profiles were also subject to multivariate regression. This analysis confirmed diabetes, cardiovascular and respiratory as differentially expressed between CSI and non‐CSI groups.

**TABLE 3:** Multivariate analysis.

Variable	*β*	95% CI	*p*
Admission type	1.07	0.99–1.16	0.08
Unplanned return to theatre	1.09	1.00–1.18	< 0.05
Surgical specialty	1.01	1.00–1.02	< 0.01
Unplanned admission to ICU	1.11	1.04–1.19	< 0.01
Comorbidities present	1.27	1.18–1.36	< 0.001

Abbreviations: CI, confidence interval; ICU: intensive care unit.

## 4 Discussion

CSIs were present in one‐third (33.3%) of surgical deaths, reflecting a considerable burden of infectious complications in the surgical population. Importantly, infections acquired during hospitalisation were more common than those present on admission, suggesting that a substantial proportion of infections are potentially preventable. These cases were associated with higher complication rates and longer lengths of stay. Patients with a CSI are a more medically complex group with a significantly higher burden of comorbidities, particularly age‐related and respiratory conditions. Reassuringly, antibiotic therapy was administered appropriately in 97.2% of cases and treatment delays were uncommon, with 90.0% of CSI cases receiving timely infection management. This points to effective infection recognition and antimicrobial stewardship within the institutions involved. However, the fact that two‐thirds of the infections acquired during admission were contracted postoperatively raises concerns about surgical site infections and other operative risks. This finding underscores the need for enhanced perioperative infection control measures, including proper wound care, aseptic techniques, prophylactic antibiotic use and earlier patient mobilisation.

This study is consistent with other studies that show the burden of infection in surgical patients is substantial, with one in 10 patients developing infection within 30 days of an operation and one in five of these patients developing multiple infections—30‐day mortality rates were more than three times higher in surgical patients with infections [5, 6]. The occurrence of infections within 30 days of surgery (most commonly surgical site infections, urinary tract infections and pneumonia) has been associated with increased incidence of mortality in surgical patients [7]. This is consistent with our study where pneumonia was the leading infection acquired during admission; patients with higher numbers of comorbidities (especially respiratory disease) were at increased risk of developing postoperative pulmonary complications, particularly if they were immobilised or ventilated. Prevention of hospital‐acquired pneumonia (HAP) and ventilator‐associated pneumonia (VAP) involves measures to minimise colonisation and aspiration as well as addressing the individual risk factors for HAP or VAP in surgical patients [8]. In addition, early recognition and appropriate antimicrobial treatment of HAP and VAP are essential for preventing prolonged hospital stay, respiratory failure and death [8, 9, 10].

General surgery accounted for close to half of the deaths in patients with a CSI in this study. Anastomotic leaks were the most common postoperative complication in patients with a CSI and are strongly linked to sepsis, prolonged recovery and mortality [11, 12, 13]. Procedure‐related sepsis was reported in over one quarter of CSI cases, further highlighting the high burden of infection‐driven complications and the potential need for more aggressive early intervention or enhanced perioperative care in this group. This is an area where early recognition, source control and appropriate antimicrobial therapy are likely to improve outcomes [11, 12, 13].

Death after urological surgery is uncommon, but in our cohort this specialty had the highest infection burden relative to case volume and the majority of urology patients (51.0%) who died had a CSI [14, 15] Previous reports from ANZASM data identified failure to administer prophylactic antibiotics or recognise urosepsis as significant preventable clinical management issues leading to mortality [16, 17, 18, 19] Urosepsis is one of the most common causes of surgical mortality, with identification of high‐risk patients and early and appropriate antimicrobial therapy essential to improve outcomes.

This study showed that patients with a CSI were less likely to undergo surgical intervention than those without a CSI. The most striking difference was observed in the timing and urgency of surgery. Patients without a CSI were more likely to be operated on within 2 h of admission, while those with a CSI were more likely to undergo surgery more than 24 h after admission. However, delay to surgery has been shown to increase the risk of acquiring CSIs [5, 20] A nationwide review of elective cases (coronary artery bypass grafts, colectomies and lung resections) in the USA found an increased risk of infectious complications and death when surgery was delayed [20] Similarly, a Danish nationwide cohort study over 10 years found that delays to surgery were associated with a significant increase in pneumonia, particularly in patients with comorbidities [5] Another reason for a delay to surgery may be failed non‐operative management of postoperative complications (e.g., anastomotic leaks or procedure‐related sepsis).

The higher comorbidity load in patients with CSIs likely contributed to their increased susceptibility to infection and may partially explain the higher rates of adverse outcomes observed in this study. Immune function is inhibited in the perioperative period, which increases the risk of CSI. Stress‐related hormones inhibit immune function and this, combined with perioperative tissue damage, blood transfusion, hypothermia, hyperglycaemia, postoperative pain and the administration of anaesthetics, greatly reduces immune function [21]. These immunosuppressive factors are enhanced in patients who are already under increased immune stress due to the presence of comorbidities [22].

Patients with CSIs experienced higher rates of adverse events, including unplanned readmissions, critical care admissions, returns to the operating theatre and postoperative complications, with anastomotic leaks being the most frequent postoperative complication. These findings underscore the significant impact of CSIs on patient outcomes, highlighting the need for effective preventive strategies and timely management to reduce the incidence of infections and associated complications.

Microbiological data from the 39.7% of CSI cases in which the infective organism was identified revealed that most CSI cases (68.1%) were caused by a single infective organism. Monomicrobial infections are often easier to treat with targeted therapy, which may have contributed to the high rate of appropriate antibiotic use observed. However, this also highlights the importance of prompt culture acquisition to guide treatment and minimise unnecessary use of broad‐spectrum antibiotics. The mainstay of managing infections is source control and appropriate antibiotic therapy.

One limitation of this study is the absence of denominator data. Since ACTASM only captures surgical mortality, the overall impact of CSIs in surgical patients is unknown. Another limitation is that data captured by ACTASM is self‐reported, which may have introduced some bias in the reporting. Despite these limitations, this study provides some important learning points for improving surgical care and reducing surgical mortality. There is evidence of an overall fall in surgical mortality rates since the inception of ANZASM, which may be partly due to reviews like this study as well as extensive educational feedback to surgeons [23].

## 5 Conclusion

Surgical patients with CSIs who died in ACT hospitals had more comorbidities, were less likely to undergo an operation, and had longer time periods between admission and operation than surgical patients without CSIs. More unplanned events (readmission to hospital or a critical care unit, return to theatre, postoperative complications) occurred in patients with CSIs. These findings underscore the significant impact of CSIs on patient outcomes, highlighting the need for effective preventive strategies and timely management to reduce the incidence of infections and associated complications.

## Conflicts of Interest

The authors declare no conflicts of interest.

## Data Availability

The data that support the findings of this study are available from the corresponding author upon reasonable request.
